# New York Letter

**Published:** 1893-06

**Authors:** 


### Correspondence.

### NEW YORK LETTER.

NEW YORK, May 14, 1893.

At a recent meeting of the Surgical Society Dr. Meyer presented an interesting case of intra-capsular fracture of the neck of the femur which he had treated by nailing the head to the neck. The patient was a man, 30 years of age, who had fractured his thigh ten months before admission to the hospital, in December of 1892. At this time examination of the injured leg showed a shortening of three and a half inches, the trochanter was higher than on the normal side, the leg was rotated outwards, on applying light and continuons traction the trochanter could be drawn down some distance, and on rotating the limb and comparing it with the normal side the trochanter was found to describe a smaller arc than normal. All the symptoms pointed to an intra-capsular fracture of the neck of the femur including the cause which was a fall in which the patient struck directly on his feet. A longitudinal incision was made over the joint and the capsule opened. The neck was found to be fractured just at the base of the head, and both the fractured end of the neck and the head presented a concave surface. The surfaces were scraped with a sharp spoon and found to bleed freely, especially the head. The head was then cut away with a chisel, the periosteum having been turned back, until it presented a convex surface to fit into the concave surface of the neck, which had been freshened by scraping and the fibrous exudate removed. The parts were then fitted together and secured by two wire nails, which were passed through the neck into the head, and a suitable dressing applied with fifteen pounds extension. When seen by Dr. Meyer, on the following morning, the patient presented symptoms of acute anæmia and approaching collapse. A subcutaneous injection of 800 c. c. and an intra venous infusion of 1000 c. c. of a saline solution were given. After this his condition improved and he had made such progress towards recovery that when presented, April 13th, he could walk readily with a cane, though with a slight limp. There was a shortening of one and a half inches which was overcome by a suitable shoe. There seemed to be firm union between the parts.

Dr. Lange made a thorough examination of the patient and concluded that there was not bone union but fibrous union of the parts as he thought he could detect a slight motion between the head and neck but the result was an excellent one as it gave the man a comparatively good limb and rendered him able to earn a living.

Dr. Lange presented two cases in which he had performed nephrectomy and in one of which he used a slightly different incision from that ordinarily employed. He used the lumbar incision but extended it higher up than usual, removing the eleventh and twelfth ribs, and his transverse incision instead of coming off at the lower part of the longitudinal was made about over the center of the kidney, thus giving him more room to work in and cutting no large vessels. He spoke of the use of the cystoscope in confirming diagnoses; in both of these cases he had used it and was able to see bloody urine coming from the ureter of the diseased side while clear urine came from that of the healthy side.

At a recent meeting of the Surgical Section of the Academy of Medicine, Dr. Coley presented a child on which he had performed Bassini's operation for strangulated hernia which ran a very unusual course. The hernia had been down twenty-four hours, the pulse was rapid and feeble, the respiration shallow, abdomen distended and tympanitic. On opening the sac about six inches of the small intestine and some omentum were found, neither of which presented any symptoms of gangrene. Immediately after the operation the child's temperature rose to 107 degrees. There were abundant mucous rales over the chest but no consolidation of the lung. The patient was put into a bath at a temperatnre of 95 degrees and kept there about fifteen minutes, after which it seemed some better, the temperature was somewhat lower and the child's general condition much improved. After a few hours the temperature again rose and the bath was repeated. This continued for three or four days, the temperature ranging from 105 to 107 degrees, after which it gradually fell and the patient made a good recovery, the side on which the hernia had been, being quite as firm as the other side. The most interesting question with regard to this case is what was the temperature due to? as it came on too quickly after the operation to have been due to acute peritonitis and there was not enough trouble in the lungs to account for it. None of the members present had any solution for the question. Dr. Dowd mentioned a similar case in which the temperature did not rise so high or remain up so long as it did in this case, and was accounted for by pneumonia of which the child presented symptoms. The cold bath is being used more and more not only in typhoid fever patients but also in the majority of diseases in which we have high temperatures to deal with.

Dr. R. T. Morris read a paper on the Pathology of Appendicitis. He described the appendix as a soft distensable tube of mucous membrane within a resisting tube of muscle and peritoneum. It is so situated that it is exposed to cold, diseases, and rheumatism, while its connection with the general alimentary canal renders it especially liable to become the receptacle of foreign bodies. He divided the pathological changes which occur in the organ into the following: 1, catarrhal inflammation; 2, infectious exudative inflammations; 3, inflammation in which the lymph channels are gorged with leucocytes and bacteria and the blood vessel walls infiltrated with the same; 4, swelling of the mucous tube; 5, thrombo-necrosis of tiny portions of the mucous tube; 6, strangulation of the mucous tube within a passive muscle tube or when the muscular tube is in a state of tonic spasm; 7, necrosis of small or large portions of the mucous tube. He presented specimens of the majority of these forms showing both the gross and microscopical pathology. He thought any inflamed appendix should be removed, and stated that when there are no complications only a very small opening need be made, large enough to introduce one finger, when the colon will appear and having made out the cord-like line along its surface all that is necessary is to push it along, following this line, and the appendix will soon appear in the opening and is easily and quickly removed, and the wound is closed, no drainage being necessary. After having worked out this admirable classification of the pathological changes, he seemed to think the little organ could not give trouble enough but that it should be assisted by the donation of a new name. To the Purist the idea of using a name derived partly from the Greek and partly from the Latin may be distasteful, but, to the large majority of practitioners, the name vermiform appendix seems perfectly satisfactory, and to replace it by the proposed name, ecphyas, would be like removing an old land mark only to replace it by one more difficult to recognize by the large majority. The length of time that the term has been in use should over-rule the objection to its having been derived from two tongues, which fact, indeed, should not make it such a bad word; the mule is not such a bad animal and yet it is as much jack as horse. One point he urged in favor of the change is that if there are other appendices than the vermiform and appendicitis, for which he would use ecphyaditis, might mean inflammation of any one of these. Yet I think that few would question as to which appendix was referred to when the term appendicitis is used without a qualifying adjective.

At a meeting of the Practitioners Society Prof. Peabody reported a most interesting case. The patient had been treated for pleurisy a year and a half ago and since that time had taken cold very easily. When admitted to the hospltal he claimed that for the last two months he had been growing short of breath and suffered from a cough with scanty, yellow sputa, occasionally streaked with blood, which he thought came from his nose. Other points in the history pointed to a probable pleurisy. There was flatness all over the left chest in front, breathing was amphoric above and the voice was exaggerated. The heart was not displaced, the heart sounds were regular and of good force but muffled. Behind over the left chest the percussion note was flat from apex to base. Breathing was amphoric from apex to angle of scapula, below which it was lost. The voice was also lost below the angle of the scapula but heard above it. The patient was in a good general condition and except for the changes in the lung gave no evidences to lead one to suspect the condition found on autopsy. Prof. Peabody did not state whether or not the chest was distended on the diseased side but I have no doubt that he took it into consideration, for, as a student under him, I have frequently heard him mention it as an important symptom. The patient died after remaining in the hospital a few days, after coughing up a small amount of blood.

On autopsy an aneurism was found pressing on the left bronchus, causing occlusion of the latter, and there was an opening from the aneurism into the bronchus with evidences of there having been a small opening before, from which the blood he had expectorated previous to coming into the hospital probably came. The left lung was completely consolidated with mottled gray and reddish areas, the bronchi contained clotted and fluid blood. This is another instance of the difficulty in diagnosing fluid in the pleural cavity of which I mentioned a number in my last letter. To the young graduate the diagnosis of pleurisy with effusion seems one of the most simple diseases to make out, but when men like Kinnicut, Peabody, Robinson, and others, say that, in some cases, it is one of the most difficult to diagnose, it becomes a serious question unless we admit the aspirating needle as one of the ordinary means of making a diagnosis.

